# Supplementary material for: An RNA-binding protein acts as a major post-transcriptional modulator in Bacillus anthracis
Source: Nat Commun. 2022 Mar 21;13:1491. doi: 10.1038/s41467-022-29209-4 (PMC8938561; doi:10.1038/s41467-022-29209-4)
Supplement: Supplementary file 1 — Supplementary Information [file 41467_2022_29209_MOESM1_ESM.pdf]

SUPPLEMENTARY INFORMATION

# An RNA-binding protein acts as a major post-transcriptional modulator in *Bacillus anthracis*

Hualiang Pi[1,2] Andy Weiss[1,2], Clare L. Laut[1,2], Caroline M. Grunenwald[1,2], Hannah K. Lin[3], Xinjie I. Yi[3], Devin L. Stauff[3], and Eric P. Skaar[1,2]

[1]Department of Pathology, Microbiology, & Immunology, Vanderbilt University Medical Center, Nashville, TN;

[2]Vanderbilt Institute for Infection, Immunology, and Inflammation, Vanderbilt University, Nashville, TN;

[3]Department of Biology, Grove City College, Grove City, PA

[#]Address correspondence to eric.skaar@vumc.org

**Supplementary Table 1. Strains and plasmids used in this study**

| Species | Genotype | Description | Reference |
|---|---|---|---|
| *B. anthracis* strain Sterne | WT | Wildtype laboratory stock | Lab stock |
| | WT $P_{hit}$*ermC* | Genetic selection | [1] |
| | Δ*krrA* $P_{hit}$*ermC* | Verifying genetic selection | This study |
| | Δ*krrA* Δ*hitRS* $P_{hit}$*ermC* | Verifying genetic selection | This study |
| | Δ*krrA* $P_{hit}$*ermC* *pOS1*$P_{lgt}$*krrA-3xFLAG* | Complementation *krrA* deletion *in trans* | This study |
| | Δ*krrA* | Lacking the *krrA* reading frame | This study |
| | Δ*krrA* pOS1.$P_{lgt}$*krrA-3xFLAG* | FLAG-tagged KrrA for immunoprecipitation | This study |
| | WT pOS1.$P_{hit}$*xylE* | XylE reporter strain | [2] |
| | Δ*krrA* pOS1.$P_{hit}$*xylE* | XylE reporter strain | This study |
| | WT pOS1.$P_{eds}$*xylE* | XylE reporter strain | [3] |
| | Δ*krrA* pOS1.$P_{eds}$*xylE* | XylE reporter strain | This study |
| | WT pOS1.$P_{hit}$*hitS*-myc | Myc-tagged HitS strain for Western blotting | This study |
| | Δ*krrA* pOS1.$P_{hit}$*hitS*-myc | Myc-tagged HitS strain for Western blotting | This study |
| *B. subtilis* strain 168 | WT | Wildtype laboratory stock | Lab stock |
| | *kre*::*erm* | Lacking the *kre* reading frame | [4] |
| | *kre*::*erm* $P_{spac}$*krrA* | Complementing *kre* deletion by expression *krrA in trans* | This study |
| *E. coli* | DH5α WT | Wildtype laboratory stock for cloning | Lab stock |
| | K1077 WT | Wildtype laboratory stock for cloning | Lab stock |
| | DH5α pET14b krrA-His | Recombinant vector for cloning | This study |
| | BL21(DE3) pREL pET14b *krrA* | Recombinant vector for protein expression | This study |
| **Plasmid** | **Description** | | **Reference** |
| pLM4 | Plasmid for gene deletion | | Lab stock |
| pET14b | Plasmid for protein expression | | Lab stock |
| pOS1 | Plasmid for complementation | | Lab stock |

## Supplementary Table 2. Oligonucleotides used in this study

| Primer name | Sequence | Use |
|---|---|---|
| *3905*_XmaI_fwd | GCATGACCCGGGCTCGCACTACCACCAATTGC | Gene deletion |
| *3905*_SOE-L | GCCAAACTGTTATTGAATCTCCTTTCAATTTTCC | |
| *3905*_SOE-R | GGAGATTCAATAACAGTTTGGCCAATTGTAGG | |
| *3905*_SacI_rev | GCATGAGAGCTCGTAAAAAAGGTGCGAGTGTGG | |
| Bas3905_NdeI_F | CCGCATATGAAACCTTCACAACCACAATC | Protein expression |
| Bas3905_BamH1_R | CGCGGATCCTTATTTTTTTGTATAAGTACGAC | |
| BAS3905-SphI | CCACGCATGCTTATTTTTTTGTATAAGTACGAC | |
| BS_kre_F | GCTGTGGAAGTGAATGTTCAG | Deletion verification |
| BS_kre_R | AAGTTGTAGGCGCAGATGAG | |
| pPL82_check_Rev | ACGATCTTTCAGCCGACTCA | Cloning |
| pPL82-check-for | AAGAAAGATATCCTAACAGCACA | |
| 5' Flag_fwd | AAATACAATTGAGGTGAACAGATTATAAGGATCATGATGGTGAT | FLAG-tagged KrrA |
| 5' Flag_rev | AAGGTTTCATACCACCACCACCACCCTTG | |
| bas3905F_5Flag | TGGTGGTGGTATGAAACCTTCACAACCAC | |
| bas3905R_5Flag | AAACACTACCCCCTTGTTTGTTTTTTTGTATAAGTACGACTATAC | |
| bas3905F_3Flag | AAATACAATTGAGGTGAACAATGAAACCTTCACAACCAC | |
| bas3905R_3Flag | CACCACCACCTTTTTTTGTATAAGTACGACTATAC | |
| 3' Flag_fwd | TACAAAAAAAGGTGGTGGTGGTGGTGATTATAAG | |
| 3' Flag_rev | AAACACTACCCCCTTGTTTGCTTGTCGTCATCGTCTTTGTAG | |
| 3' Flag_gblock | GGTGGTGGTGGTGGTGATTATAAGGATCATGATGGTGATTATA | |
| 5' Flag_gblock | GATTATAAGGATCATGATGGTGATTATAAGGATCATGATATCGA | |
| BAS3905_fwd | CTGCTAAATAGCCGTAACGTGC | Cloning |
| BAS3905_rev | GTATGACAGCTATAATAAATCG | |
| ermC_qPCR_fwd | TGAAATCGGCTCAGGAAAAGG | qPCR |
| ermC_qPCR_rev | GGAAATTTAAACTGCAATATATCCTTG | |
| BAS_16S_rRNA_F | GAAGGCGACTTTCTGGTCTG | |
| BAS_16S_rRNA_R | CCTTTGAGTTTCAGCCTTGC | |
| BAS5200_qPCR_fwww | TTTTGCTCCTGCCATAAGCC | |
| BAS5200_qPCR_rev | CGCCCTGGATACTTTGAACG | |
| BAS1815_qPCR_fw | TTGTTACGGTGTTGAGGGGG | |
| BAS1815_qPCR_rev | ATGGCAATTCGCTGCCTTTG | |
| BAS1816_qPCR_fw | CTAGTAGTGAGGGTTAAGGCGT | |
| BAS1816_qPCR_rev | CGAACAAGTTCTCCCCGCTT | |
| BAS_hssR_qPCR_fw | CGGGCAGACGATTTTGCTTC | |
| BAS_hssR_qPCR_rev | TGAGAAACGATCTCGGAGCC | |
| hitP_probe_R | GCTTAGCATTTGTGACTTTCACACCAG | Northern blotting |
| hitR_probe1_R | CGAGTTCTCTAATATGCGGATCATCGTC | |
| hitR_probe2_R | GGTAACGTAACCGTTTGTTCTCCAATTG | |
| hitS_probe_R | CTTTCTTGGTCAGCAGTTATATGCAC | |
| 16S_probe_R | CCTTTGAGTTTCAGCCTTGCGGCC | |
| HitRCDS_T7_F | TAATACGACTCACTATAGGGATGAATAATCAGTGGGGATTGT | EMSA |
| HitRCDS_rev | AACCATATCAACTTTCACTTG | |

**Supplementary Table 3. Isolated spontaneous erythromycin resistant suppressors with frameshift mutations in *bas3905/krrA.***

| Background | Suppressor* | Allele | Genotype |
|---|---|---|---|
| WT $P_{hit}$*ermC* | C11 | *bas3905*-1 | Transversion at nucleotide 114 (T → A) → frameshift after amino-acid residue 38 |
| | B26, C16, C17, E11, E32, E47, E50 | *bas3905*-2 | Deletion of A at nucleotide 126 → frameshift after amino-acid residue 44 |
| | E30 | *bas3905*-3 | Transversion at nucleotide 137 (C → A) → frameshift after amino-acid residue 45 |
| | C22, D38 | *bas3905*-4 | Insertion of A at nucleotide 356 → frameshift after amino-acid residue 121 |
| | B28, C12, C14, C15, C18, C19, E21, E26, E27, E31 | *bas3905*-5 | Deletion of A at nucleotide 357 → frameshift after amino-acid residue 121 |

Suppressor*, letters denote individual suppressors isolated from different batches of genetic selections.

**Supplementary Table 4. KrrA-interacting RNA targets located in intergenic regions**

| | | | | Input RNA control (reads) | | Untagged control (reads) | | KrrA-FLAG vehicle (reads) | | KrrA-FLAG ‘205 (reads) | |
|---|---|---|---|---|---|---|---|---|---|---|---|
| # | start | stop | locus | Rep. #1 | Rep. #2 | Rep. #1 | Rep. #2 | Rep. #1 | Rep. #2 | Rep. #1 | Rep. #2 |
| 1 | 2168991 | 2169040 | BAS_11225-BAS_11230 | 9 | 10 | 12 | 12 | 3126 | 3467 | 4390 | 7212 |
| 2 | 3125310 | 3125364 | BAS_16060-BAS_16065 | 7 | 3 | 33 | 22 | 2339 | 2409 | 2878 | 4013 |
| 3 | 1999197 | 1999260 | BAS_10370-BAS_10375 | 7 | 20 | 17 | 34 | 1810 | 1793 | 2159 | 2162 |
| 4 | 241860 | 241900 | BAS_01325-BAS_01330 | 61 | 80 | 56 | 40 | 1345 | 1299 | 1615 | 1579 |
| 5 | 799816 | 799903 | BAS_04190-BAS_04195 | 3 | 4 | 4 | 9 | 1026 | 1058 | 1221 | 1254 |
| 6 | 4500446 | 4500482 | thpR-pepV | 0 | 1 | 1 | 1 | 244 | 244 | 300 | 340 |
| 7 | 2368639 | 2368704 | BAS_12190-BAS_12195 | 2 | 2 | 5 | 15 | 211 | 186 | 318 | 407 |
| 8 | 3100452 | 3100510 | BAS_15920-BAS_15925 | 0 | 2 | 6 | 4 | 131 | 151 | 152 | 244 |
| 9 | 1150160 | 1150222 | BAS_05935-BAS_05940 | 0 | 1 | 0 | 0 | 101 | 104 | 95 | 139 |
| 10 | 2578634 | 2578680 | BAS_13235-BAS_13240 | 0 | 0 | 0 | 0 | 97 | 108 | 127 | 159 |
| 11 | 3088773 | 3088820 | BAS_15860-BAS_15865 | 1 | 2 | 1 | 1 | 92 | 128 | 129 | 104 |
| 12 | 3164063 | 316114 | BAS_16250-BAS_16255 | 0 | 0 | 2 | 0 | 85 | 129 | 96 | 26 |
| 13 | 1835492 | 1835564 | BAS_09450-BAS_09455 | 0 | 0 | 0 | 0 | 84 | 62 | 77 | 93 |
| 14 | 4123275 | 4123321 | BAS_RS21365-BAS_RS21370 | 0 | 1 | 1 | 0 | 55 | 33 | 54 | 56 |
| 15 | 2864017 | 2864048 | BAS_15095-BAS_15100 | 2 | 1 | 0 | 0 | 51 | 48 | 55 | 49 |
| 16 | 2332898 | 2332957 | BAS_12025-BAS_12030 | 1 | 0 | 0 | 1 | 24 | 29 | 32 | 33 |
| 17 | 2832187 | 2832237 | BAS_14635-BAS_14640 | 0 | 0 | 3 | 1 | 18 | 23 | 23 | 15 |
| 18 | 2263145 | 2263189 | BAS_11675-BAS_11680 | 2 | 2 | 0 | 0 | 15 | 19 | 14 | 13 |
| 19 | 2173480 | 2173532 | BAS_11255-BAS_11260 | 0 | 0 | 0 | 1 | 12 | 15 | 15 | 18 |

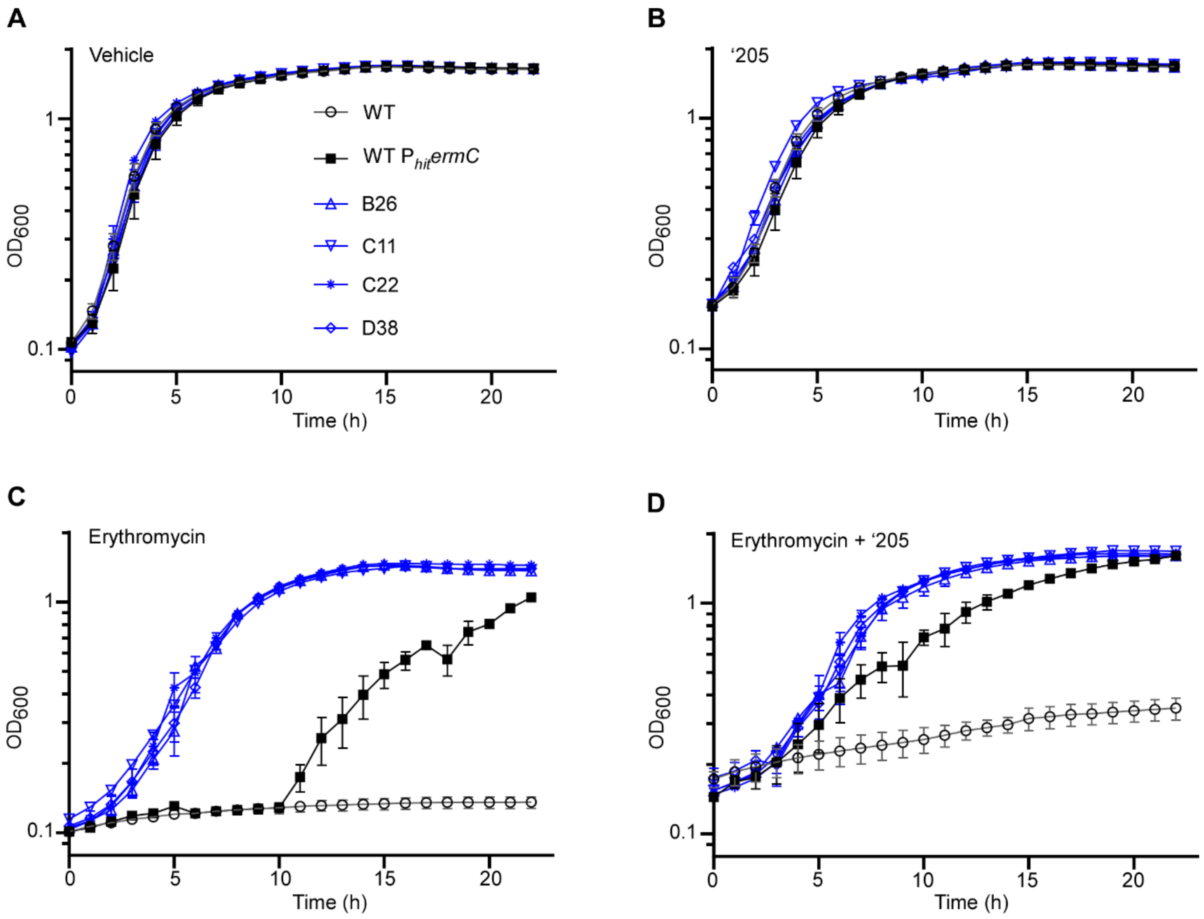


**Supplementary Fig. 1. Isolated spontaneous erythromycin resistant suppressors with frameshift mutations in *krrA*.** Growth kinetics of *B. anthracis* WT, WT $P_{hit}$*ermC*, and representative suppressors were monitored for 24 h in vehicle (**A**), 20 µM ‘205 (**B**), 2 µg ml$^{-1}$ erythromycin (**C**), or 20 µM ‘205 plus 2 µg ml$^{-1}$ erythromycin (**D**). The experiments were performed three independent times that showed similar results and data shown are averages of three biological replicates (mean ± SD).

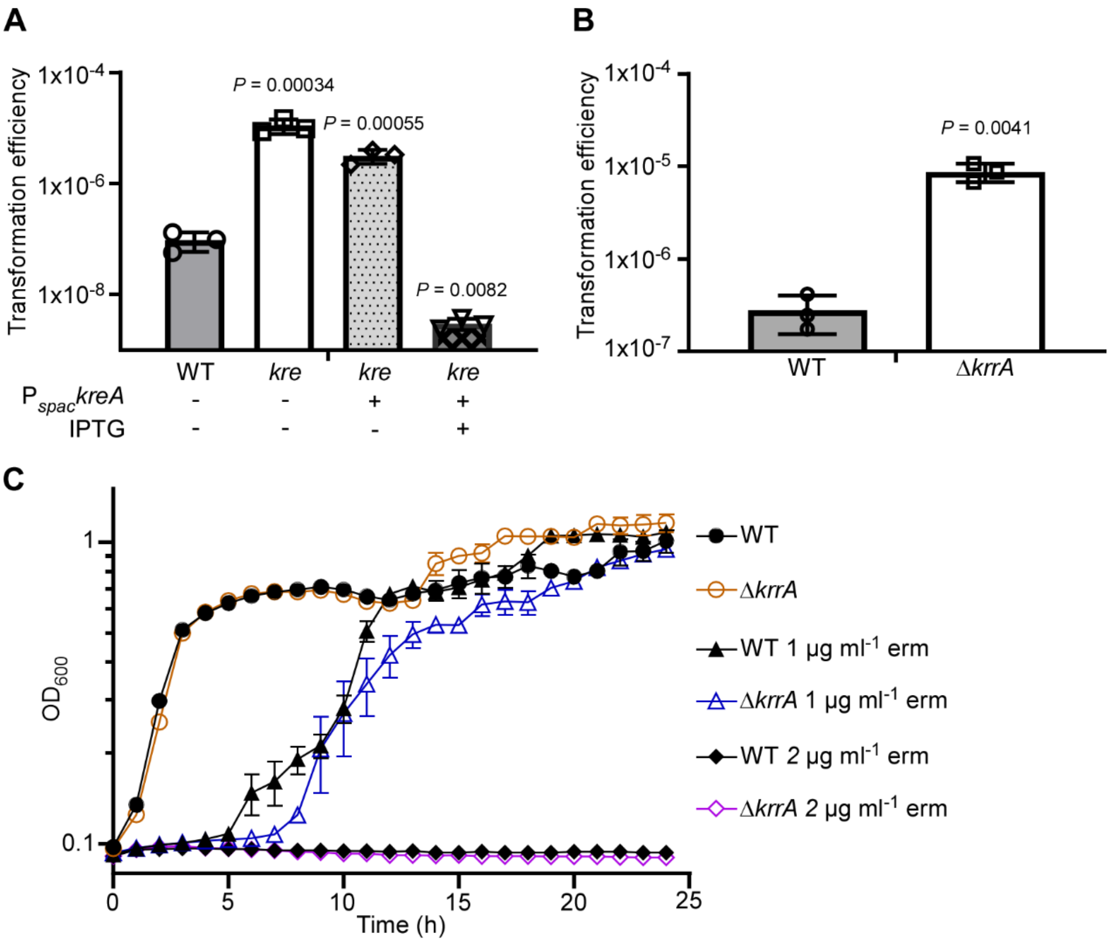


**Supplementary Fig. 2. *B. anthracis* KrrA is involved in genetic competence but plays no role in erythromycin resistance.** (**A**) Transformation efficiency of *B. subtilis* WT, *kre*::kan, and *kre*::kan expressing *B. anthracis krrA*. 1mM IPTG was used to induce expression of *krrA*. (**B**) Transformation efficiency of *B. anthracis* WT and Δ*krrA*. Data shown are the average from three independent experiments (mean ± SEM). Significant differences are determined by two-tailed *t*-tests. Source data are provided as a Source Data file. (**C**) Growth kinetics of *B. anthracis* WT and Δ*krrA* in vehicle, 1 µg $ml^{-1}$, or 2 µg $ml^{-1}$ erythromycin was monitored for 24 h. No significant differences of erythromycin resistance were observed between these two strains. The experiments were performed three independent times that showed similar results and data shown are averages of three biological replicates (mean ± SD).

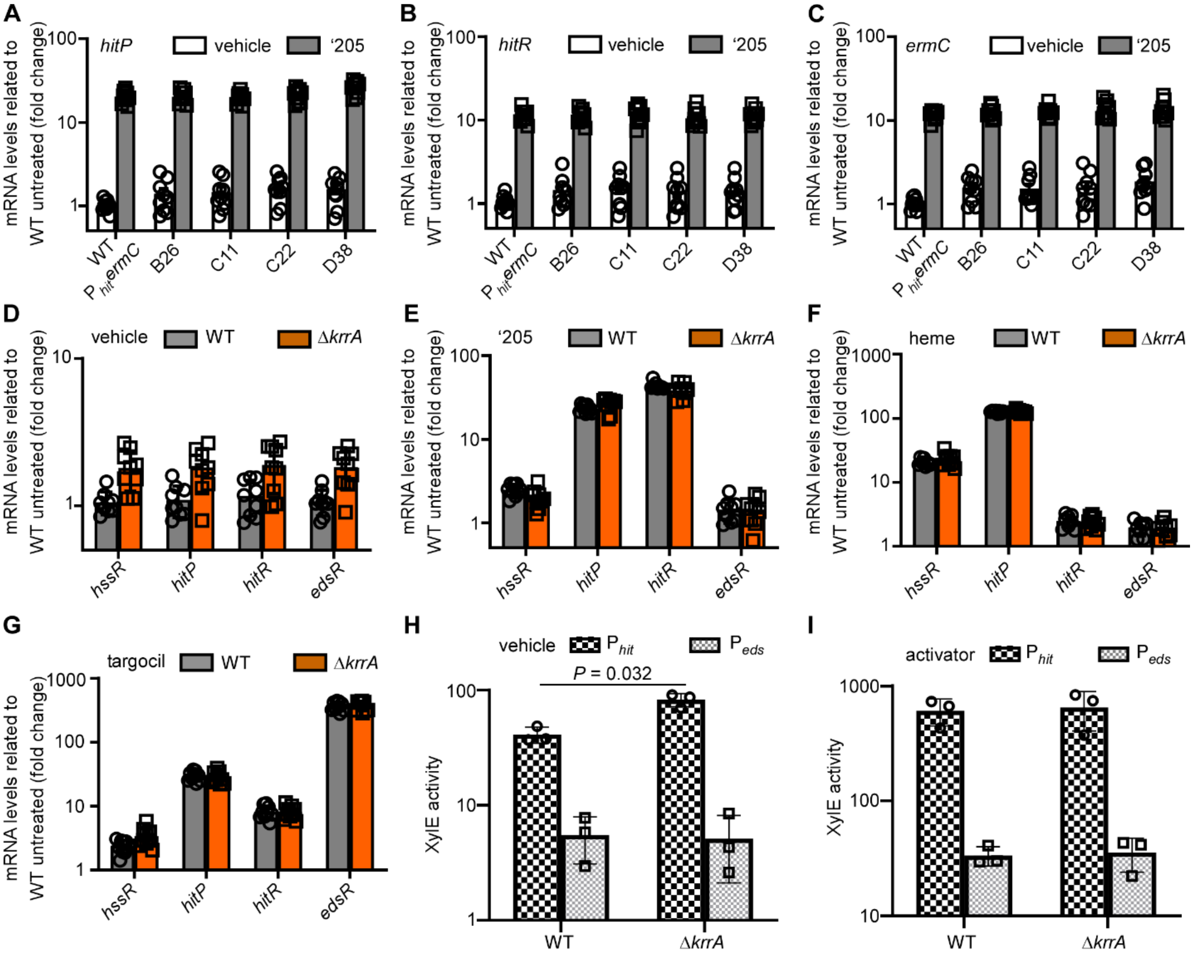


**Supplementary Fig. 3. Effects of *krrA* mutation on gene transcription.** (**A-C**) To evaluate the effects of spontaneous *krrA* mutations on transcription of *hitP* (**A**), *hitR* (**B**), and *ermC* (**C**), qPCR was performed in WT $P_{hit}$*ermC* (used for genetic selection) and four representative suppressor mutants. (**D-G**) To evaluate the effects of *krrA* deletion on transcription of TCS genes, qPCR was carried out in WT and Δ*krrA*. Genes tested were three response regulator genes (*hssR*, *hitR*, and *edsR*) and *hitP* that encodes an uncharacterized HitR-regulated transporter. Three different TCS activators were examined: 20 µM '205 (HitRS activator, **E**), 1 µM heme (HssRS activator, **F**), and 1 µg $mL^{-1}$ targocil (EdsRS activator, **G**). For all qPCR experiments, overnight cultures were inoculated with a 1:100 ratio into fresh LB medium in the absence (vehicle) or presence of treatment as indicated. After 6 h of vigorous shaking at 37°C, cells were harvested, and total RNA was extracted and subjected to cDNA synthesis followed by qPCR quantification. *B. anthracis* 16S rRNA was used as a housekeeping internal control. The experiments were performed three independent times with three biological replicates each time. The data are expressed as the mean ± SEM (n=9). (**H-I**) To further examine the effects of *krrA* deletion on TCS activation, activity of $P_{hit}$ and $P_{eds}$ was quantified in WT and Δ*krrA* carrying a XylE reporter. Briefly, overnight cultures were inoculated with a 1:100 ratio into fresh LB medium in the absence (vehicle) or presence of TCS-specific activator (20 µM '205 for HitRS and 1 µg $mL^{-1}$ targocil for EdsRS). After 6 h of vigorous shaking at 37°C, XylE activity was quantified and normalized to bacterial optical density. The experiments were performed three independent times that showed similar results and the representative data are expressed as the mean ± SD (n=3). Significant differences are determined by two-tailed t-tests. Source data are provided as a Source Data file.

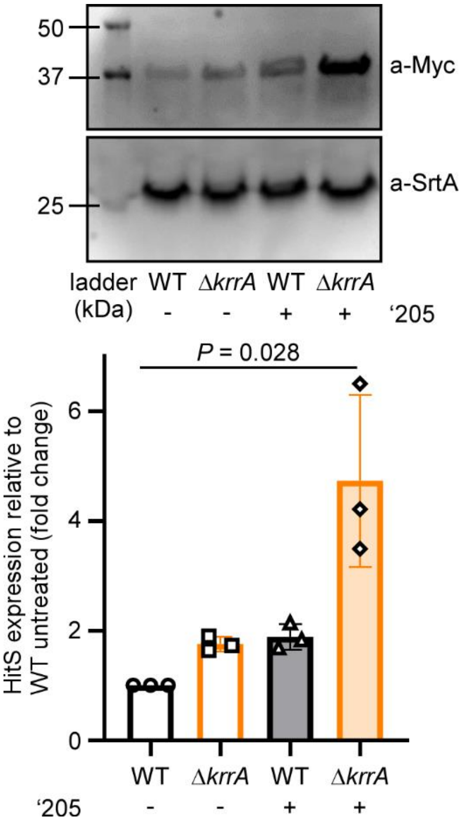


**Supplementary Fig. 4. Effects of KrrA-mediated RNA regulation on HitS expression.** Western blot analysis of HitS levels was carried out in WT and Δ*krrA* strains harboring pOS1.P$_{hit}$*hitS-myc* with or without '205 treatment. The Myc-tagged HitS were detected using an anti-Myc antibody. Anti-SrtA serum was served as a normalization control. Data in the bottom panel are averages of three independent experiments (mean ± SEM). Significant differences are determined by two-tailed t-tests. Source data are provided as a Source Data file.

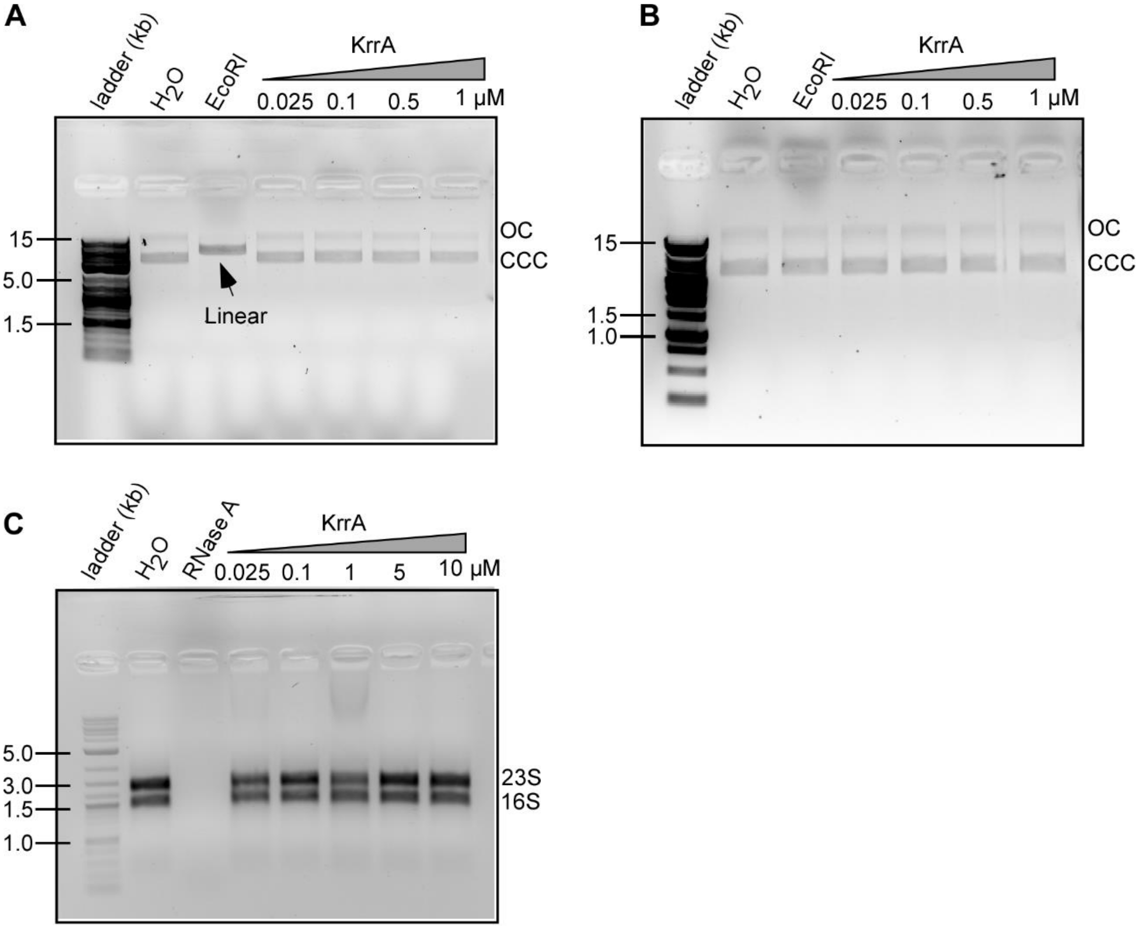


**Supplementary Fig. 5. KrrA has no detectable nuclease activity.** (**A** and **B**) DNA endonuclease activity of KrrA was tested in CutSmart buffer (**A**) or phosphate buffer (**B**). No detectable DNA endonuclease activity was observed with up to 1 μM of the purified KrrA protein used. (**C**) RNA nuclease activity of KrrA was tested in phosphate buffer. No evident RNA nuclease activity was observed with up to 10 μM of KrrA tested. The experiments were performed three independent times that showed similar results and representative data are shown.

## References

1 Pi, H. *et al.* Directed evolution reveals the mechanism of HitRS signaling transduction in *Bacillus anthracis*. *PLoS Pathog* **16**, e1009148, doi:10.1371/journal.ppat.1009148 (2020).

2 Mike, L. A. *et al.* Two-Component System Cross-Regulation Integrates *Bacillus anthracis* Response to Heme and Cell Envelope Stress. *PLOS Pathogens* **10**, e1004044, doi:10.1371/journal.ppat.1004044 (2014).

3 Laut, C. L. *et al.* *Bacillus anthracis* Responds to Targocil-Induced Envelope Damage through EdsRS Activation of Cardiolipin Synthesis. *mBio* **11**, e03375-03319, doi:10.1128/mBio.03375-19 (2020).

4 Mašlaňová, I., Stříbná, S., Doškař, J. & Pantůček, R. Efficient plasmid transduction to *Staphylococcus aureus* strains insensitive to the lytic action of transducing phage. *FEMS Microbiol Lett* **363**, doi:10.1093/femsle/fnw211 (2016).
