# Supplementary material for: An RNA-binding protein acts as a major post-transcriptional modulator in Bacillus anthracis
Source: Nat Commun. 2022 Mar 21;13:1491. doi: 10.1038/s41467-022-29209-4 (PMC8938561; doi:10.1038/s41467-022-29209-4)
Supplement: Supplementary file 2 — Reporting Summary [file 41467_2022_29209_MOESM2_ESM.pdf]

nature portfolio

Corresponding author(s): Eric P Skaar

Last updated by author(s): Feb 23, 2022

Reporting Summary

Nature Portfolio wishes to improve the reproducibility of the work that we publish. This form provides structure for consistency and transparency in reporting. For further information on Nature Portfolio policies, see our Editorial Policies and the Editorial Policy Checklist.

Statistics

For all statistical analyses, confirm that the following items are present in the figure legend, table legend, main text, or Methods section.

n/a	Confirmed	
[ ]	[x]	The exact sample size (n) for each experimental group/condition, given as a discrete number and unit of measurement
[ ]	[x]	A statement on whether measurements were taken from distinct samples or whether the same sample was measured repeatedly
[ ]	[x]	The statistical test(s) used AND whether they are one- or two-sided<br>*Only common tests should be described solely by name; describe more complex techniques in the Methods section.*
[x]	[ ]	A description of all covariates tested
[ ]	[x]	A description of any assumptions or corrections, such as tests of normality and adjustment for multiple comparisons
[ ]	[x]	A full description of the statistical parameters including central tendency (e.g. means) or other basic estimates (e.g. regression coefficient) AND variation (e.g. standard deviation) or associated estimates of uncertainty (e.g. confidence intervals)
[ ]	[x]	For null hypothesis testing, the test statistic (e.g. F, t, r) with confidence intervals, effect sizes, degrees of freedom and P value noted<br>*Give P values as exact values whenever suitable.*
[x]	[ ]	For Bayesian analysis, information on the choice of priors and Markov chain Monte Carlo settings
[x]	[ ]	For hierarchical and complex designs, identification of the appropriate level for tests and full reporting of outcomes
[x]	[ ]	Estimates of effect sizes (e.g. Cohen's d, Pearson's r), indicating how they were calculated

Our web collection on statistics for biologists contains articles on many of the points above.

Software and code

Policy information about availability of computer code

Data collection	No software was used to collect data for this study.
Data analysis	Bioinformatic analysis of the RNA sequencing data was performed using CLC genomic workbench software (version 20.0.1).

For manuscripts utilizing custom algorithms or software that are central to the research but not yet described in published literature, software must be made available to editors and reviewers. We strongly encourage code deposition in a community repository (e.g. GitHub). See the Nature Portfolio guidelines for submitting code & software for further information.

Data

Policy information about availability of data

All manuscripts must include a data availability statement. This statement should provide the following information, where applicable:
- Accession codes, unique identifiers, or web links for publicly available datasets
- A description of any restrictions on data availability
- For clinical datasets or third party data, please ensure that the statement adheres to our policy

The RNA-seq and RIP-seq data that support the findings of this study have been deposited in GEO repository with the accession number GSE193212. The RNA-seq dataset can be found in this link https://www.ncbi.nlm.nih.gov/geo/query/acc.cgi?acc=GSE193210 and the RIP-seq dataset can be found in this link https://www.ncbi.nlm.nih.gov/geo/query/acc.cgi?acc=GSE193211. Source data are provided with this paper.

Field-specific reporting

Please select the one below that is the best fit for your research. If you are not sure, read the appropriate sections before making your selection.

- [x] Life sciences
- [ ] Behavioural & social sciences
- [ ] Ecological, evolutionary & environmental sciences

For a reference copy of the document with all sections, see nature.com/documents/nr-reporting-summary-flat.pdf

Life sciences study design

All studies must disclose on these points even when the disclosure is negative.

Sample size	No statistical method was used to predetermine sample size, but we routinely employed at least three biological replicates for each experiment. The sample size for each figure is indicated in figure legends.
Data exclusions	No data were excluded from analysis.
Replication	All experiments were performed independently at least three times, sometimes even more when some experiments failed due to technical issues.
Randomization	Experimental groups were formed according to genotype, where necessary, or randomized, where possible.
Blinding	This study did not involve any experimental subject group; therefore, experimenter binding did not apply and was not performed.

Reporting for specific materials, systems and methods

We require information from authors about some types of materials, experimental systems and methods used in many studies. Here, indicate whether each material, system or method listed is relevant to your study. If you are not sure if a list item applies to your research, read the appropriate section before selecting a response.

Materials & experimental systems

n/a	Involved in the study	
[ ]	[x]	Antibodies
[x]	[ ]	Eukaryotic cell lines
[x]	[ ]	Palaeontology and archaeology
[x]	[ ]	Animals and other organisms
[x]	[ ]	Human research participants
[x]	[ ]	Clinical data
[x]	[ ]	Dual use research of concern

Methods

n/a	Involved in the study	
[x]	[ ]	ChIP-seq
[x]	[ ]	Flow cytometry
[x]	[ ]	MRI-based neuroimaging

Antibodies

Antibodies used	The antibodies used were Anti-Flag M2 monoclonal antibody (Sigma-Aldrich, cat # F1804), α-myc (Abcam, cat # ab9132), and α-SrtA rabbit serum that was developed from a prior study.
Validation	All antibodies used in this study were validated either by the company that sold them or published literature. Further validation by us included the use of bacterial strains lacking the target antigen.
