# Supplementary material for: An RNA-binding protein acts as a major post-transcriptional modulator in Bacillus anthracis
Source: Nat Commun. 2022 Mar 21;13:1491. doi: 10.1038/s41467-022-29209-4 (PMC8938561; doi:10.1038/s41467-022-29209-4)
Supplement: Supplementary file 4 — Description of Additional Supplementary Files [file 41467_2022_29209_MOESM4_ESM.pdf]

Title: Supplementary Data 1
Description: RNA targets of KrrA identified by RIPseq under vehicle or '205-treated (50 µM) conditions. The fold enrichment cutoff is ≥ 3 and the P-value cutoff is < 0.05.

Title: Supplementary Data 2
Description: transcriptomic comparison between Bacillus anthracis ΔkrrA and WT under vehicle or '205-treated (50 µM) conditions. Genes with significantly differential expression in ΔkrrA relative to WT are shown. The fold change cutoff is ≥ 3 and the P-value cutoff is < 0.05.

Title: Supplementary Data 3
Description: transcriptomic comparison between '205 and vehicle treatments of Bacillus anthracis W T. Genes significantly upregulated or downregulated following '205 treatment (50 µM) are shown. T he fold change cutoff is ≥ 3 and the P-value cutoff is < 0.05.

Title: Supplementary Data 4
Description: the broad effects of KrrA-mediated RNA regulation and its coregulatory gene networks revealed by comparisons between fRIP-seq and RNA-seq datasets. 50 µM '205 were used where indicated. The fold change cutoff is ≥ 3 and the P-value cutoff is < 0.05.
